# Dynamic variation of nutrient absorption, metabolomic and transcriptomic indexes of soybean (*Glycine max*) seedlings under phosphorus deficiency

**DOI:** 10.1093/aobpla/plad014

**Published:** 2023-04-10

**Authors:** Mingxia Li, Ji Zhou, Qi Liu, Lili Mao, Haoru Li, Shuying Li, Rui Guo

**Affiliations:** School of Life Sciences, ChangChun Normal University, Changchun 130024, China; Land Consolidation and Rehabilitation Centre, The Ministry of Land and Resources, Beijing 100035, China; Institute of Environment and Sustainable Development in Agriculture, Chinese Academy of Agricultural Sciences, Beijing 100081, China; Institute of Environment and Sustainable Development in Agriculture, Chinese Academy of Agricultural Sciences, Beijing 100081, China; Institute of Environment and Sustainable Development in Agriculture, Chinese Academy of Agricultural Sciences, Beijing 100081, China; Forestry and Grassland Bureau of Aohan Banner, Chifeng City 024000, InnerMongolia; Institute of Environment and Sustainable Development in Agriculture, Chinese Academy of Agricultural Sciences, Beijing 100081, China

**Keywords:** *Glycine max*, metabolomic, phosphorus deficiency, roots, transcriptomic

## Abstract

The dynamic trajectory of metabolites and gene expression related to phosphorus absorption and utilization in soybean seedling roots were determined under short- and long-term phosphorus deficiency stress. The metabolome results showed that *TCA* and *GS/GOGAT* cycles were enhanced after 2 days of phosphorus deficiency stress; however, they were inhibited after 15 days. GC-TOF-MS showed that phosphorus deficiency increased the accumulation of amino acids significantly after 2 days, whereas organic acids and lipid substances increased significantly after 15 days. Quantitative reverse transcription-polymerase chain reaction (RT-PCR) showed that transcriptional levels of five key genes related to phosphorus activation and phosphorus starvation signal transduction increased continuously with phosphorus deficiency. The expression of *GmPHT1* and *GmSPX* triggered the phosphorus starvation signal pathway and induced the expression of the *GmPS* and *GmPAP* genes to enhance the synthesis and secretion of organophosphorus hydrolase and organic acid in soybean roots under phosphorus deficiency. The phospholipid metabolism was enhanced significantly after 15 days of stress and when *GmSQD*, a crucial enzyme in lipid biosynthesis, was up-regulated. Thus, we propose that future investigations on stress caused by phosphorus deficiency should include more organs obtained at different developmental stages.

## Teaser

In our study, the dynamic trajectories of metabolites and gene expression related to phosphorus uptake and utilization in soybean seedling roots under phosphorus deficiency were determined by comparing growth parameters, mineral nutrient contents, metabolism and transcription levels at different growth and development stages. The study identified metabolites, metabolic pathways and genes that play a key role in short- and long-term phosphorus deficiency stress in soybean. This study can provide important reference values for improving soybean stress resistance.

## Introduction

Soybean (*Glycine max*) is one of the most important economic and oil-bearing crops worldwide, and 30 % of the global production of edible oil and 69 % of dietary protein are derived from it (Zhao *et al*. 2019). It contains various nutritional and health-promoting compounds such as high-quality protein, unsaturated fatty acid, calcium, and B vitamins, which are believed to have various health-promoting effects (Li *et al*. 2017; Li *et al*. 2019). In addition, soybean can adapt to a broad range of environmental conditions, such as saline-alkaline soils (Shi *et al*. 2016; Zhang *et al*. 2016a). Therefore, soybean has been used many times to study the molecular mechanisms of resistance to adversity (Fu *et al*. 2020).

Phosphorus (P) is a nutrient necessary for plant growth and nutrient accumulation (Lynch 2011). In nature, very little P is available to plants in soils due to the adsorption of metal cations and the activities of microorganisms (Cordell and White 2015; Wang *et al*. 2018b). An intensive application of P has become a standard agricultural practice to ensure crop productivity; but excessive use of P fertilizer causes the eutrophication of water bodies (Zhang *et al*. 2016b). Exploring the molecular mechanisms of crop adaptation to P deficiency in soil is an important issue in agricultural research from the perspective of the dynamic relationship between soil and crops.

Roots are the link between plants and soil, and also the organ that allows plants to absorb nutrients and water. They play an essential role in the physiological activities of plants. Therefore, understanding the mechanism of P adaptability in the root is conducive to an in-depth understanding of plant survival and growth, and the ecological adaptation strategies to P-deficient environments (Hermans *et al*. 2006; Lynch 2011; Lambers *et al*. 2015). Studies have shown that under P deficiency, the phenotypic structure of roots changes, secondary metabolism and the metabolites exuded by roots are altered, and genes associated with changes in root architecture are expressed (Péret *et al*. 2014; Wang *et al*. 2018a). Thus, it is feasible to reveal the relationship between crops and soil from the perspective of the roots.

Many studies have demonstrated that plants will produce reactive oxygen under P deficiency, which can lead to membrane lipid peroxidation and membrane damage (Rouached *et al*. 2010). However, plants have evolved various adaptive strategies for P efficiency, including changes in morphology, physiology adaptations, and biochemical and molecular processes (Bucher *et al*. 2001). Consequently, the stress in response to P deficiency leads to the degradation of phospholipids to obtain P for plant use, while sulfolipids and galactolipids are synthesized to replace phospholipids and maintain membrane stability (Zhang *et al*. 2019). Previous studies provide a theoretical basis and new ideas for the study of the metabolome and molecular mechanisms underlying the response of soybean roots to P deficiency.

In this study, high-throughput sequencing technology and integrated transcriptomic and metabolomic analyses were used to compare differences in the nutrient elements and metabolic pathways of small-molecule metabolites and gene expression in soybean seedling roots cultivated for 1, 2, 6 or 15 days under P deficiency stress. The objectives of this study were to identify the dynamic trajectory of DEGs in soybean seedling roots under P deficiency, particularly up-regulated transcripts associated with P signalling and transporting, and to determine the changes in metabolites caused by changes in gene expression. The overall objective was to identify metabolites, metabolic pathways and genes that play crucial roles in soybeans in response to short-term and long-term P deficiency stress.

## Methods

### Plant growth and stress treatments

The seeds of soybean plants (*Glycine max*) were sown in 14-cm-diameter plastic pots containing 2.5 kg of washed sand and all pots were placed outdoors. The temperatures were 18.5 ± 1.5 °C at night and 25 ± 2 °C during the day and the relative humidity was 60 ± 5 %. Four seeds of a single material were planted per pot and one seedling in each pot was retained. After 5 weeks, 30 pots were divided randomly into five groups, which included one control group and four groups under different times of P deficiency stress. The P-deficient stress groups were irrigated with a Hoagland nutrient solution with 2.5 μM KH_2_PO_4_, and the K was supplemented with KCl at the same concentration. The control group was irrigated with a complete Hoagland nutrient solution with 2000 μM KH_2_PO_4_. The P-deficient stress was carried out from July 1 to July 16 in 2021 (Table 1).

**Table 1.** The concentration of phosphorus solutions and different stress times and the days of P-deficient stress for soybean (DS).

Treatment	P concentration (μmol·L^−1^)	Start date	End day	Treated days
CK	2000	—	16 July	0
1DS	2.5	15 July	16 July	1
2DS	2.5	14 July	16 July	2
6DS	2.5	10 July	16 July	6
15DS	2.5	01 July	16 July	15

### Growth parameter analysis

After the P-deficient stress, seedlings were separated from the sand, washed with ultra-pure water, the root nodules and length were recorded. The fresh weights of roots were recorded and then the dry weights were determined after drying for 15 min in an oven at 80 °C and then in a vacuum dryer at 40 °C to a constant weight.

### Quantification of nutrient elements

The dry samples (50 mg) were digested with HNO_3_ under 95 °C, and then diluted with water to a final volume of 10 mL, the quantities of nutrient elements were assayed using an ICP-OES spectrometer (iCAP6000 series, Thermo Fisher Scientific Inc., USA).

### Metabolite profiling analysis

The freeze-dried sample (20 mg) was placed into 2-mL sterile Eppendorf tubes (Eppendorf China Limited, Shanghai City, China). In total, 1000 μL of methanol/dH_2_O (3:1, v/v) and 10 μL ribitol (0.5 mg mL^−1^) as an internal standard were added. The tubes were then vortexed and mixed homogenized at 35 Hz for 4 min and sonicated for 5 min in an ice-water bath, by the method of metabolite extraction described by Garcia and Barbas (2011). They were then centrifuged at 12 000*g* and 4 °C for 10 min, and the supernatants were collected. The treated extract was dried in a vacuum concentrator for 30 min. Methoxyamination hydrochloride (120 μL) was added to the supernatant for amination. The samples were then incubated in an 80 °C oven for 30 min, and 120 μL of silanization reagent was added at 70 °C for 1.5 h for derivatization. After the temperature was lowered to room temperature, all samples were analysed by gas chromatography–time-of-flight mass spectrometer (GC-TOF-MC; 30 m × 250 μm; film thickness, 0.25 μm; J&W Scientific, Folsom, CA, USA), using a DB-5MS capillary column. All measurements were repeated three times with four biological replicates per sample. The injection, transfer line and ion source temperatures were 280, 280 and 250 °C, respectively (Kind *et al*. 2009). The energy was −70 eV in electron impact mode.

The raw data were acquired and pre-processed using the Chroma TOF (V 4.3x, LECO, St. Joseph, MI, USA) software and the LECO-Fiehn Rtx5 database, the peaks detected in less than half of the QC samples or relative standard deviation (RSD) >30 % in quality control samples were removed (Dunn *et al*. 2011). FiehnLib and the commercial EI-MS libraries were used to identify metabolites. Afterwards, features with at least an 80 % missing value were removed. In the following, the missing values were replaced with a small value, which was half the minimum positive value in the original data. And then, the data were filtered by interquartile range (IQR). Then, the total mass of the signal integration area was normalized for each sample. Next, a principal component analysis (PCA), partial least squares discriminant analysis (PLS-DA), orthogonal partial least squares discriminant analysis (OPLS-DA) and loading plot were performed by SIMCA-P 13.0 software package (Umetrics, Umea, Sweden) using normalized data. Variable importance values (VIP) were obtained through PLS-DA and OPLS-DA analyses. In addition, differential metabolites were found using Student’s *t-*test (*P* < 0.05) and VIP (VIP > 1) combined with similarity values greater than 700. The metabolite pathways were identified by using KEGG (http://www.genome.jp) and the MetaboAnalyst website (www.metaboanalyst.ca/; Xia *et al*. 2012).

### Transcriptome analysis

Total RNA and genomic DNA were extracted using a Spectrum™ Plant Total RNA Kit (Sigma-Aldrich, St Louis, MO, USA) and On-Column DNase I Digest kit (Sigma-Aldrich), respectively. RNA concentration was measured using NanoDrop 2000 (Thermo). RNA integrity was assessed using the RNA Nano 6000 Assay Kit of the Agilent Bioanalyzer 2100 system (Agilent Technologies, CA, USA). A total of 1 μg RNA per sample was used as input material for the RNA sample preparations. Sequencing libraries were generated using an NEB Next Ultra RNA Library Prep Kit for Illumina (NEB, Ipswich, MA, USA) following the manufacturer’s recommendations, and index codes were added to attribute sequences to each sample. Briefly, mRNA was purified from total RNA using poly-T oligo-attached magnetic beads. Fragmentation was carried out using divalent cations under an elevated temperature in NEB Next First-Strand Synthesis Reaction Buffer (5×). First-strand cDNA was synthesized using random hexamer primer and M-MuLV Reverse Transcriptase. Second-strand cDNA synthesis was subsequently performed using DNA polymerase I and RNase H. Remaining overhangs were converted into blunt ends using exonuclease and polymerase. After adenylation of 3ʹ ends of DNA fragments, the NEB Next Adaptor with hairpin loop structure was ligated to prepare for hybridization. To select cDNA fragments that were preferentially 240 bp in length, library fragments were purified with the AMPure XP system (Beckman Coulter, Beverly, CA, USA). Then 3 μL USER Enzyme (NEB) was used with size-selected, adaptor-ligated cDNA at 37 °C for 15 min followed by 5 min at 95 °C before PCR. Then PCR was performed with Phusion High-Fidelity DNA polymerase, Universal PCR primers, and Index (X) Primer. Finally, PCR products were purified (AMPure XP system) and library quality was assessed on the Agilent Bioanalyzer 2100 system. The clustering of the index-coded samples was performed on a cBot Cluster Generation System using a TruSeq PE Cluster Kit v4-cBot-HS (Illumina) according to the manufacturer’s instructions. After cluster generation, the library preparations were sequenced on an Illumina HiseqXten platform and paired-end reads were generated. After the original number, to ensure that Reads were of high enough quality to ensure the accuracy of subsequent analysis, strict quality control was carried out on the data, and the following filtering methods are carried out: (i) Remove Reads containing connectors; (ii) Remove low-quality Reads (including Reads with N removal ratio greater than 10 %; remove Reads with alkali base with mass value *Q* ≤ 10 accounting for more than 50 % of the whole Read). High-quality clean data were obtained after the above series of quality control.

The clean data were then mapped to the reference Williams 82 genome sequence based on good comparison efficiency. HISAT2 is an efficient comparison system from RNA sequencing experiment reads, the successor of TopHat2/Bowtie2. In this project, HISAT2 software is used to compare Clean Reads with the reference genome. Before comparison, the reference genome needs to be indexed first, and then run HISAT2 for comparison (HISAT2, version: 2.0.4, main parameters: -- dta - p 6 -- max-intron 5000000); Then use StringTie to compare and assemble the reads on. StringTie is an algorithm based on the optimization theory. It uses the comparison information to build a multi-variable clipping map and builds a traffic network to assemble the reads according to the maximum traffic algorithm (StringTie, version 1.3.4d, main parameters: -- merge - F 0.1 - T 0.1). By comparing the efficiency, that is, the percentage of Mapped Reads on the reference genome in Clean Reads, we could evaluate whether the selected reference genome assembly can meet the needs of information analysis.

In the process of differential expression gene detection, the Count value is used for differential analysis through DESeq2 (DESeq2, version: 1.6.3, main parameters: default: test = ‘Wald’, fitType = ‘parametric’), and the Fold Change ≥ 2 and false discovery rate (FDR) < 0.05 are used as the screening criteria. The gene that meets this condition is the differential gene of the CK and LP stress groups of both experimental materials. The fold change represents the ratio of expression between two samples (groups). The FDR is obtained by correcting the *P*-value of the difference significance.

FPKM = cDNA fragments/mapped fragments (millions) × transcript length (kb) (Florea *et al*. 2013).

Gene function was annotated based on the GO (Gene Ontology) database. The GO enrichment analysis of the differentially expressed genes (DEGs) was implemented using the GOseq R package based on a Wallenius non-central hyper-geometric distribution (Young *et al*. 2010). The DEGs related to metabolism were identified based on the Kyoto Encyclopedia of Genes and Genomes (KEGG) (http://www.genome.jp).

### Quantitative real-time PCR validation

We randomly selected 10 DEGs for real-time RT-PCR analysis to verify the reliability of the transcriptome analysis data. The real-time RT-PCR analysis was performed on a Roche Light Cycler 480 (Yang *et al*. 2016). Actin was used as an internal gene, and the relative expression was calculated by the comparative cycle threshold method (Schmittgen and Livak 2008). The specificity of the primers was assessed by TB tools software, and the primer sequences are listed in **Supporting Information—Table S1**.

### Statistical analysis

SPSS 18.0 was used for the statistical analyses of the data for the plant morphology and ions; the means followed by different letters in the same stress type are significantly different at <0.05 according to Duncan’s method.

## Results

### Growth parameters in response to P-deficient stress

**Figure 1.**
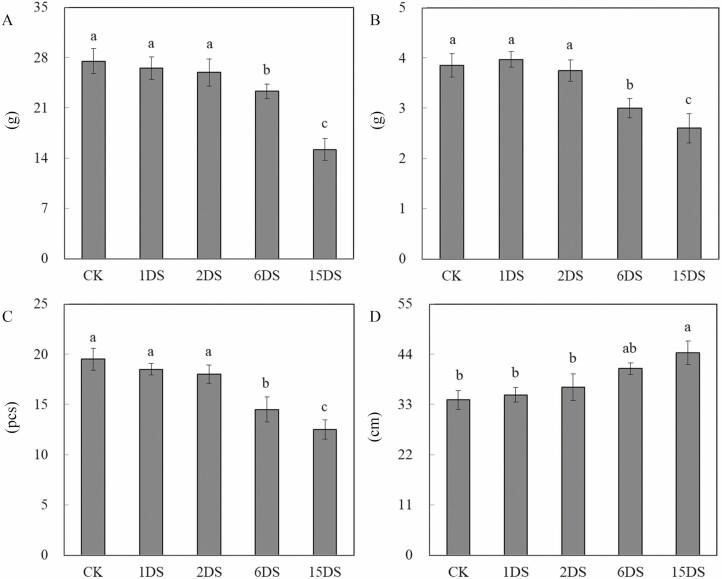
Growth performance in the roots of soybean under different levels of P deficiency, including the (A) fresh weight of roots; (B) dry weight of roots; (C) root nodule numbers; (D) root length. Data are means ± SD of three biological replicates and different small letters represent significant differences at *P* < 0.05 by the one-way ANOVA test.

As shown in Fig. 1, the dynamics of fresh/dry weight of roots and the number of root nodules were similar under P-deficient stress: these growth parameters started to decrease after 2 days, with greater reductions at 15 days, as compared with CK (Fig. 1A–C, *P* < 0.05). However, the root length was enhanced by P deficiency stress, increasing by 30.3 % after 15 days of stress (Fig. 1D, *P* < 0.05).

### Nutrient elements and free anions in response to P deficiency stress

Under P deficiency stress, the P element content decreased sharply with increasing time as compared with CK (Table 2, *P* < 0.05). The change in the trends of K, Na, Ca, Mg, Cu, Fe, Zn and Mn was similar and could be described as the V-type. The lowest value appeared after 2 days of stress or 6 days of stress, while the highest value occurred after 15 days of stress (Table 2, *P* < 0.05).

**Table 2.** The contents of nutrient elements in the roots of soybeans under P deficiency stress. Data are means ± SD of three biological replicates and different small letters represent significant differences at *P* < 0.05 by the one-way ANOVA test.

Treatment	Nutrient elements (μmol·g^–1^)								
	P	K	Na	Ca	Mg	Cu	Fe	Zn	Mn
CK	291.49 ± 24.93^a^	629.26 ± 22.35^b^	55.52 ± 4.02^a^	56.23 ± 5.20^ab^	187.85 ± 14.30^b^	0.53 ± 0.04^a^	0.12 ± 0.01^a^	0.07 ± 0.01 ^b^	0.03 ± 0.00^b^
1DS	236.55 ± 21.33^a^	514.45 ± 32.71^b^	34.22 ± 3.98^b^	49.36 ± 4.81^b^	126.17 ± 12.04^b^	0.29 ± 0.05^b^	0.07 ± 0.01^b^	0.04 ± 0.00^c^	0.02 ± 0.00^b^
2DS	160.17 ± 18.29^ab^	382.97 ± 15.88^c^	34.97 ± 4.55^b^	41.08 ± 3.72^b^	85.45 ± 7.35^c^	0.16 ± 0.02^c^	0.08 ± 0.00^b^	0.04 ± 0.00^c^	0.01 ± 0.00^b^
6DS	161.99 ± 20.64^ab^	371.69 ± 20.03^c^	19.60 ± 3.64^c^	41.37 ± 4.33^b^	135.10 ± 10.90^b^	0.26 ± 0.02^b^	0.07 ± 0.00^b^	0.03 ± 0.00^c^	0.01 ± 0.00^b^
15DS	129.17 ± 14.90^b^	845.84 ± 19.23^a^	43.37 ± 4.43^ab^	88.11 ± 3.42^a^	282.57 ± 18.82^a^	0.46 ± 0.08^a^	0.13 ± 0.02^a^	0.13 ± 0.01^a^	0.06 ± 0.01^a^

### Metabolite profiling in response to P deficiency stress

**Figure 2.**
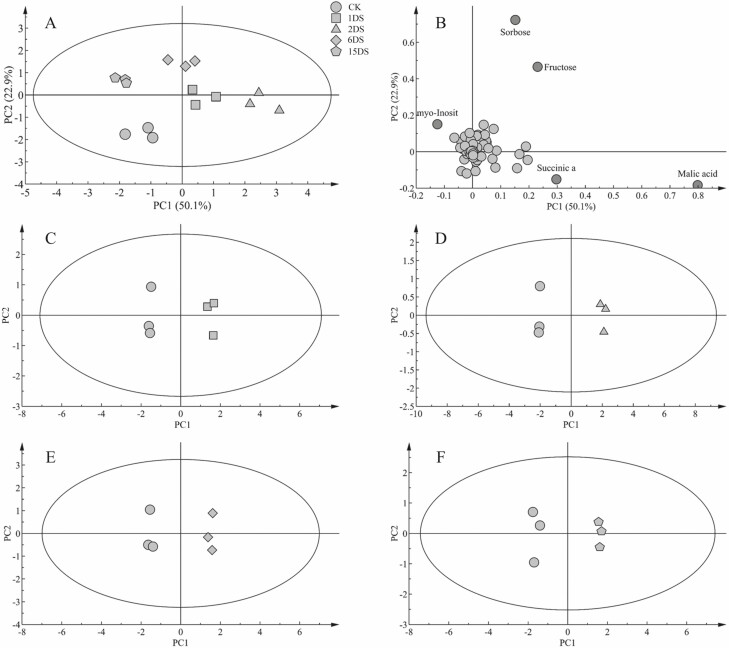
The principal component analysis (PCA) and orthogonal partial least squares discrimination analysis (OPLS-DA) of metabolic profiles and loading plots of metabolites in roots of soybean. (A) PCA of soybean; (B) loading plot of soybean; OPLS-DA between CK and (C) 1DS; (D) 2DS; (E) 6DS; and (F) 15DS.

PCA results showed that samplings were distributed within the 95 % confidence interval of Hotelling’s *T*^2^ ellipse. PC1 and PC2 indicated significant differences in the stress response between different levels of P deficiency (Fig. 2A). The results showed that malic acid, succinic acids, fructose and GABA greatly contributed to PC1, whereas sorbose, fructose, malic acid and myo-inositol greatly contributed to PC2 (Fig. 2B). The score plots of the OPLS-DA results showed a good model quality (Fig. 2C–F).

A total of 61 types of metabolites were identified, which were sugars/polyols, amino acids, organic acids, fatty acids and lipid substances. Compared with the CK, 6, 18, 7 and 27 metabolites were significantly affected by P deficiency at 1DS, 2DS, 6DS and 15DS, respectively (Table 3, *P* < 0.05). Under P deficiency, the levels of *TCA* cycle intermediates significantly increased at 1DS and 2DS, especially at 2DS; however, they decreased dramatically at 15DS (Table 3, *P* < 0.05). Even though the levels of glycolysis intermediates changed under P deficiency, this was not significant except for a significant decrease in glucose-6-phosphate at 15DS (Table 3, *P* < 0.05). In addition, substances associated with phospholipid metabolism were significantly increased at 15DS (Table 3, *P* < 0.05). The *Gs/GOGAT* cycle was enhanced at 2DS, as shown by increases in aspartic acid and asparagine, which were inhibited at 15DS (Table 3, *P* < 0.05). Under P deficiency, most amino acids increased at 2DS but decreased at 15DS (Table 3, *P* < 0.05). In general, the levels of some substances in sugars/polys and organic acids increased significantly under P deficiency (Table 3, *P* < 0.05). Most lipid substances significantly increased at 15DS, whereas they decreased at 6DS (Table 3, *P* < 0.05).

**Table 3.** The fold change of metabolites in the roots of soybeans after P deficiency. The fold change of metabolites was calculated by the ratio between the treatment and the control (*n* = 3). * indicates significance (*P* < 0.05).

Metabolite pathway and names		Fold changes			
		Log_2_^(1DS/CK)^	Log_2_^(2DS/CK) )^	Log_2_^(6DS/CK) )^	Log_2_^(15DS/CK) )^
TCA cycle	Malic acid	0.47*	0.80*	0.15	−0.79*
	Succinic acid	−0.15	0.47*	−0.03	−0.36*
	Citric acid	−0.19	0.65*	−0.20	0.33
	Fumaric acid	0.74*	1.22*	0.29	−0.63*
	Oxalic acid	−0.09	−0.13	−0.28	−0.18
	α-ketoglutaric acid	−0.07	0.86*	−0.57	−1.51*
	γ-aminobutyric acid	−0.07	0.29	0.02	0.03
Glycolysis	Pyruvic acid	−0.46	0.29	0.10	0.64
	Glucose-6-phosphate	0.03	0.13	−1.01*	−2.38*
	Fructose	0.46*	0.50*	0.65*	−0.19
	Glucose	0.33	0.07	0.21	−0.31
Phospholipid metabolism	Serine	−0.20	1.12*	0.55	0.61*
	Glycine	−0.23	0.21	0.47	0.72*
	Ethanolamine	0.07	0.42	-0.36	0.45
	myo-Inositol	0.07	0.15	0.11	0.63*
GS/GOGAT cycle	Aspartic acid	0.55	1.38*	0.46	−1.19*
	Asparagine	0.93	1.01*	0.87	−1.31*
Amino acids	Alanine	−0.12	0.01	−0.10	−0.44*
	Proline	0.17	0.47*	−0.05	−0.86*
	Valine	−0.07	0.50*	0.33	0.32
	Isoleucine	0.11	0.60*	0.44	0.22
	Threonine	0.60	1.30*	0.67	0.30
Sugars and polys	Sorbose	0.49*	0.55*	0.77*	0.07
	Maltose	−0.24	0.23	0.11	0.99*
	Ribose	−0.09	0.36	−0.21	0.49
	Talose	0.44	0.16	0.34	−0.21
	Xylitol	−0.33	0.11	−0.10	0.55
	Levoglucosan	−0.12	0.21	0.48	0.66
	Xylose	0.05	0.57	0.51	0.52
	Mannitol	−0.53	0.29	−0.17	0.82*
	Galactinol	0.28	1.07*	1.58*	0.43
	Trehalose	−0.42	0.24	−0.21	0.81*
	Raffinose	0.31	0.56	0.33	0.03
	Cellobiose	−0.13	0.02	−0.42	−0.23
	Fucose	1.30*	1.88*	1.01*	−0.40
	Mannose	0.37	0.70	0.45	0.14
	Lyxose	0.16	0.78	0.79	0.73
Organic acids	Lactic acid	−0.35	0.12	−0.54	0.68*
	Valeric acid	0.26	0.70*	0.17	0.71*
	Malonic acid	1.50*	1.69*	0.37	−0.67
	Benzoic acid	−0.23	0.05	−0.07	0.64
	Shikimic acid	−0.27	−0.09	−0.16	0.27
	Galactonic acid	−0.28	0.14	0.38	1.20*
	Glycolic acid	0.21	0.31	0.15	0.21
	Mucic acid	−0.03	-0.63	−0.34	1.30*
	Maleic acid	0.22	0.53	−0.15	−0.87
	Threonic acid	0.31	0.53	0.28	1.05*
	Gluconic acid	−0.06	0.16	0.26	1.25*
	Lactobionic acid	−0.50	0.09	−0.42	0.45
	Citramalic acid	−0.02	0.30	−0.24	−0.01
	Pelargonic acid	0.36	0.38	0.32	0.20
Lipid substances	Palmitic acid	−0.11	−0.01	−0.34	0.81*
	Glycerol	0.01	−0.03	−0.40	0.35
	Stearic acid	−0.31	−0.18	−0.70*	0.14
	Glyceric acid	−0.11	0.49	0.12	0.61*
	Monopalmitin	0.08	0.47	−0.07	0.71*
	Linoleic acid	−0.43	0.35	−0.53	0.75*
	Linolenic acid	−0.52	0.34	−0.85*	0.84*
	Myristic acid	−0.44	0.09	−0.43	0.20
	Gluconiclactone	-0.54	−0.26	0.10	0.28
	Monostearin	−0.04	0.17	−0.12	0.72*

### Differences in transcript profiles in response to P deficiency stress

**Figure 3.**
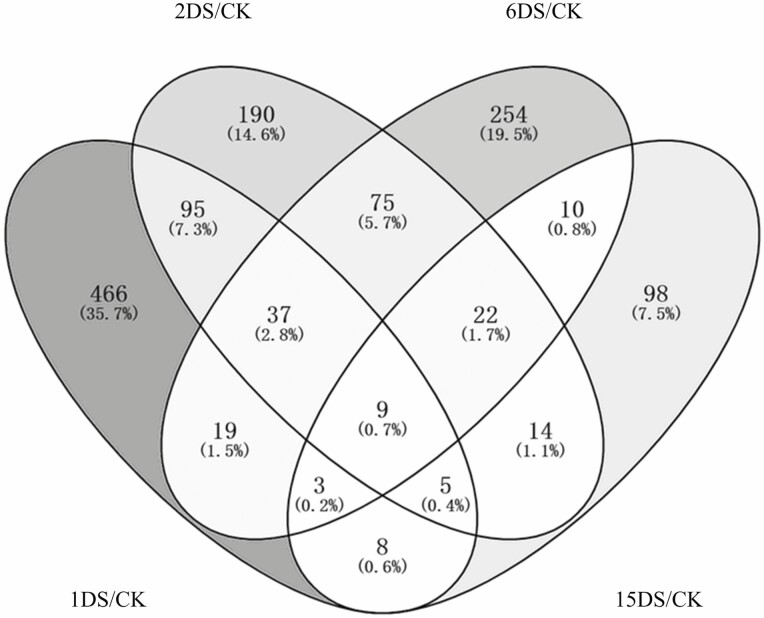
Overlap of differentially expressed genes (DEGs) generated from different treatments. The shade of colour represented the proportion of genes to total DEGs.

**Figure 4.**
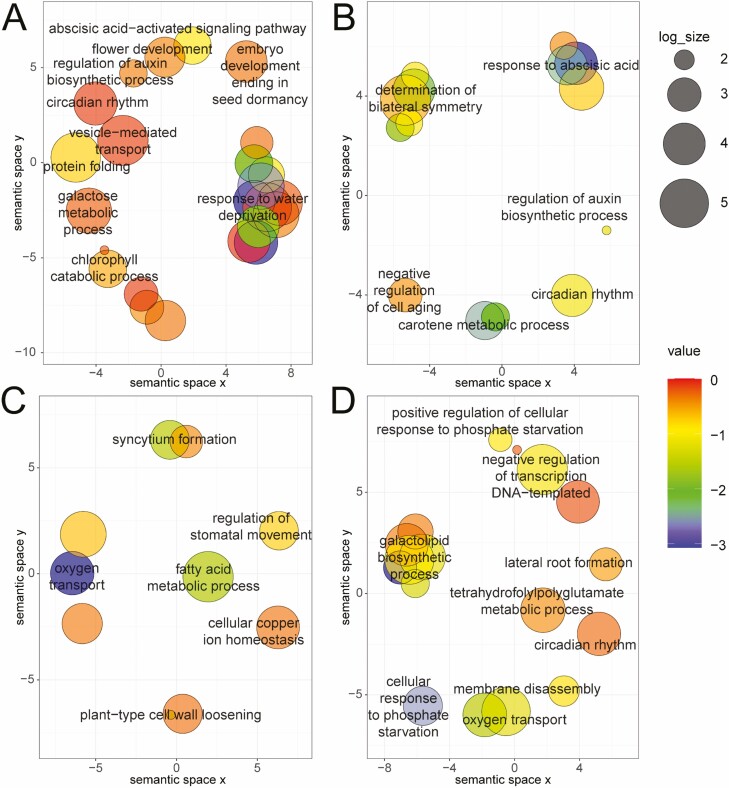
Gene Ontology (GO) enrichment analysis. A significant enriched GO term was calculated by an R package cluster Profiler and summarized by REVIGO (http://revigo.irb.hr/). Bubble colour indicates the *P*-value; size indicates the frequency of the GO term in the underlying GOA database (bubbles of more general terms are larger).

The qRT-PCR results for 10 genes in the roots of soybean were consistent with the expression found in the transcriptome data, verifying the accuracy of the transcriptome data **[see****Supporting Information—Fig. S1****]**. RNA-seq analysis was used to compare the difference in the expression of P-tolerant-related genes between CK and different levels of P deficiency. Under P deficiency, 642, 447, 429 and 169 DEGs were identified at 1DS, 2DS, 6DS and 15DS, respectively, as compared with CK (Fig. 3). To understand the functions of different transcription patterns, GO enrichment analyses were performed (Fig. 4). As a result, DEGs involved in short-term P deficiency stress (1DS and 2DS) were mainly involved in circadian rhythm and ABA-signalling transcription changes (Fig. 4). However, in mid-term (6DS) and long-term (15DS) P deficiency stress, DEGs were enriched in ion homeostasis and development, such as ‘cellular copper ion homeostasis’, ‘plant-type cell wall loosening’, ‘cellular response to phosphate starvation’ and ‘lateral root formation’ (Fig. 4).

### Differences in the expression of genes to P deficiency stress

**Figure 5.**
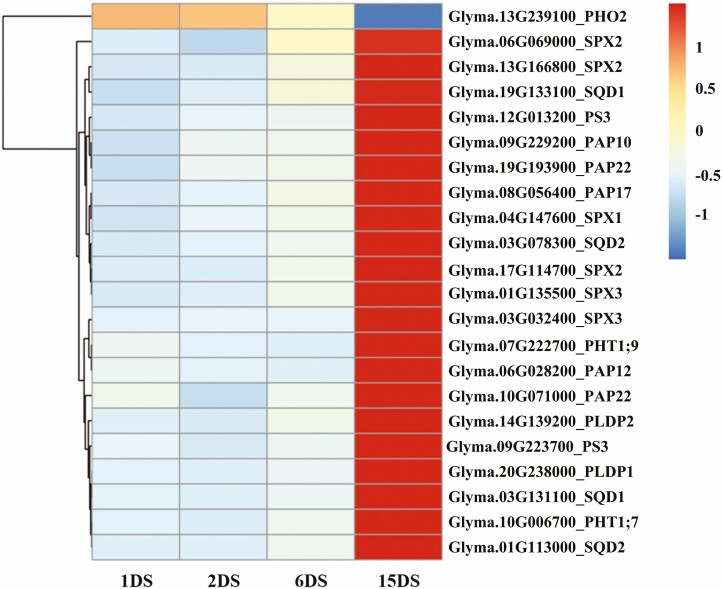
Gene expression of representative phosphorus deficiency-related genes. Colour represents the *Z*-score normalized TPM value of genes.

To further investigate the function of important genes in the P starvation response, we extracted the expression pattern of genes with function validation in soybeans. Most genes were down-regulated after short-term P deficiency stress, whereas only the *GmPHO2* gene was up-regulated (Fig. 5). At 15 days of P deficiency stress, the expression of 21 genes from P metabolism-related gene families in the roots of soybean was up-regulated significantly including one *GmPS3*, two *GmPHT*, two *GmPLDP*, four *GmPAP*, three *GmSPX* and two *GmSQD*, and only the *GmPHO2* gene was down-regulated (Fig. 5).

## Discussion

### Growth parameters in response to P deficiency stress

Stress caused by P deficiency severely affects different organs or tissues in the plant causing limitations in plant growth and productivity (Xu *et al*. 2012). Growth parameters can reflect the condition of the plant and are considered a useful index in determining the degree of stress. Under P deficiency stress, the fresh and dry weight of soybean decreased, indicating that low P might interfere with the uptake of elements in the roots, and the injurious effect of P deficiency stress was severe after 6 days. The injurious effects of P deficiency stress are commonly thought to be a result of low mineral nutrients, and P deficiency induces severe reductions in membrane stability by decreasing levels of phospholipids (Vance *et al*. 2003). However, the root lengths of soybean increased under P deficiency stress.

### Nutrient elements in response to different P deficiency stress

Under P deficiency, not only is the absorption and accumulation of P limited but also the accumulation, transport and metabolism of other elements are affected (Schlüter *et al*. 2013). In this study, the content of P decreased due to P deficiency at 2 days, and a decrease was also observed at 15 days (Table 2, *P* < 0.05). These findings showed that soybean maintained relatively stable P content under P deficiency over 2 days. Low Na and high K concentrations in soybean are normally essential to maintaining metabolic processes (Zhu 2003). In addition, K and Mg are important elements of abiotic stress; they facilitate the proper functioning of many enzymes and their normal activity catalyses important physiological processes, especially in membrane stability. Our results showed that K and Mg declined and began to rise as time passed, a phenomenon perhaps related to the plasma membrane, which may be one of the mechanisms of self-defence. Moreover, with the extension of time, the changes in nutrient elements and free anions reflected the effects of P deficiency on metabolism and indicated that the physiological responses of plants to long-term P deficiency stress were more complex than that of short-term stress.

Ca acts as a mineral regulatory element that protects plants from stress and plays an irreplaceable role in alleviating abiotic stress (Lin *et al*. 2014). In addition, during P deficiency stress, Ca plays an important role in regulating *CAX1* and *CAX3* protein kinase pathway-mediated expression, which affects P uptake and accumulation (Liu *et al*. 2011). In this study, long-term P deficiency increased Ca accumulation in soybean roots, and the increase of Ca could activate miR399-*PHO2* signalling to increase P efficiency and diminish the damage to plants caused by P deficiency. In this study, compared with the CK, long-term P deficiency stress led to the accumulation of Cu, Fe, Zn and Mn to maintain and protect the function of the organism.

### Metabolite profiling in response to P deficiency stress

Understanding the metabolomic and molecular mechanisms underlying root responses to P deficiency stress in soybean is important for sustainable staple leguminous plant production and optimizing future production. The *TCA* cycle plays an important role in the source of energy for cells. In this study, we found that the *TCA* cycle was dramatically enhanced after short-term P deficiency, but significantly inhibited after long-term deficiency. In short-term P deficiency, enhancement of the *TCA* cycle was beneficial for maintaining a stable energy supply for the growth of soybean; moreover, intermediate products were produced and accumulated by the *TCA* cycle such as amino acids (Sha *et al*. 2016; Li *et al*. 2022). The results suggested that soybean roots might consume energy to promote the biosynthesis of amino and organic acids that were compatible solutes during short-term P deficiency stress. However, the long-term low availability of P in the soil largely inhibited energy metabolism causing a relatively unstable supply of material and energy to resist P deficiency stress, limiting the growth of the soybean plant. In the *GS/GOGAT* cycle, aspartic acid and asparagine levels increased after short-term P deficiency stress, suggesting that these changes in the amounts of transamination-related metabolites are consistent with the shift of metabolic activities towards proline biosynthesis.

Regulating the distribution of sugars/polys can provide resources for the growth and development of plants to adapt to P deficiency (Hammond and White 2008; Li *et al*. 2022). In this study, some sugars/polys were increased more with the increasing length of stress, suggesting that they could be used for energy metabolism and complex carbohydrate biosynthesis, regulating soybean growth and development to adapt to P deficiency stress. The levels of organic acids increased significantly after long-term P deficiency stress, which might help reduce the consumption of P during the phosphorylation of sugar metabolites and convert small phosphorylated metabolites into sugars.

Phospholipid metabolism is one of the most important secondary metabolic pathways in plants that can produce lipid substances to maintain the integrity and stability of the cell membrane structure, and enhance plant stress resistance (Palma *et al*. 2008; Zhang and Rock 2008). This present study showed that the level of lipid substances increased significantly after long-term P deficiency stress but decreased less during short-term stress. The results implied that a high content of lipid substances was crucial for roots to develop low P stress tolerance, and active synthesis metabolism was a basic response for roots to tolerate low P stress.

### Differences in transcript profiles in response to P deficiency stress

P plays an indispensable role in the processes of the plant life cycle. The uptake and transport of P in plants involve multiple signalling molecules such as transcription factor regulation, miRNA regulation and inter-organelle transfer regulation (Puga *et al*. 2017; Wang *et al*. 2018a). *PHO2* has been identified as a pivotal signalling cascade protein involved in the phosphate starvation response and ensures the stability of the *PHT* protein to maintain the normal absorption and transport of P (Lin *et al*. 2009; Oono *et al*. 2013). The results showed that the expression of *GmPHO2* was down-regulated, whereas *GmPHT1;7* and *GmPHT1;9* were up-regulated by P deficiency, which implied that the *GmPHT* genes could be downstream components of *GmPHO2*. *SPX* proteins are critical for the P signalling pathway and regulate P homeostasis in plants. In addition, they are known as the central regulators of the absorption of other nutrients under abiotic stress (Yao *et al*. 2014). The expression of three *GmSPX* members (*GmSPX1*, *GmSPX2*, and *GmSPX3*) was up-regulated by P deficiency stress, suggesting that *GmSPX* is a positive regulator in the P signalling network, and controls P homeostasis in soybean. The expression levels of GmSPX members were the same as previous results in other plant species under P deficiency stress, such as AtSPX1, AtSPX2 and AtSPX3 in Arabidopsis, and OsSPX1, OsSPX2 and OsSPX3 in rice (Duan *et al*. 2008; Wang *et al*. 2009b).

It has previously been reported that the over-expression of the PAP gene family led to a significant improvement in P efficiency in cell-wall proteomic analysis of soybean roots (Wang *et al*. 2009a). In this study, we identified soybean purple acid phosphatase (*GmPAP10*, *GmPAP12*, *GmPAP17* and *GmPAP22*) as being a major *ATPase*, and its over-expression could significantly improve the utilization efficiency of organic P in soybean. In this study, the expression of the soybean phosphate starvation-induced gene 3 (*GmPS3*) and phospholipase D P (*GmPLDP1*, *GmPLDP2*) decreased during early P efficiency stress, while it increased in soybean roots with the increasing duration of P deficiency, implying *GmPS3* and *GmPLDP1* gene expression may be a part of the long-term P deficiency response mechanism. Sulfoquinovosyl diacylglycerol (*SQD*) is a key enzyme, which is associated with lipid biosynthesis, and its gene expression level has a significant positive correlation with adversity (Zhan *et al*. 2017). Our results showed that *SQD1* and *SQD2* were up-regulated specifically during the stress associated with long-term P deficiency stress. This indicated that the phospholipid metabolites that regulated the synthesis of lipid substances were promoted to participate in membrane lipid remodelling to ensure the integrity of the cell membrane system.

## Conclusions

The damage to plants from P deficiency depends on the type, intensity and duration. Maintaining P homeostasis is critical for plant growth and environmental responses. It is important to understand the mechanism of tolerance to P deficiency stress in plants to improve the efficiency of soil P. In this study, after 15 days of P deficiency stress, the root length was increased to some extent compared with the control, suggesting that soybeans increased the contact area between root and soil to promote the ability of the root to absorb P. The level of P and nutrient elements showed that compared with control samples, the P content in the roots of soybean decreased with the duration of P deficiency, whereas nutrient elements levels increased under long-term stress, which could efficiently compensate for the P deficiency to ensure the basic P demand for growth and development. The metabolome detected a total of 61 metabolites in roots, which could be classified into 12 categories. Under different times of P deficiency, the amino acids, sugars/polys, organic acids and lipid substances in the roots of soybean changed, but the change in trends were not the same. We confirmed that long-term low P availability in soil largely inhibited energy metabolism and the *GS/GOGAT* cycle, whereas phospholipid metabolism was enhanced to decompose phospholipids and release lipid substances for reuse and to resist P deficiency stress. Moreover, prolonged P deficiency induced the progressive accumulation of organic acids to improve resistance to P deficiency stress. P uptake and transport in soybean plants involve multiple signalling molecules that mainly include P activation and P starvation signal transduction. The up-regulation of *GmPS*, *GmPHT1*, *GmPAP*, *GmSPX* and *GmSQD* under P deficiency deserves further attention. This experiment provided a reference for the study of soybean adaptation to P deficiency through the regulation of metabolites and molecules and new insights for soybean resource evaluation.

## Supporting Information

The following additional information is available in the online version of this article—

**Table S1.** Primers for qRT-PCR of the10 genes in the roots of soybean.

**Figure S1.** The qRT-PCR of 10 genes in the roots of soybean.

plad014_suppl_Supplementary_Figure_S1

plad014_suppl_Supplementary_Table_S1

## Data Availability

The raw sequence data of transcriptomic were deposited in the Genome Sequence Archive (https://bigd.big.ac.cn/gsa) under accession number subCRA006201. The raw data of metabolomic were deposited in the metabolights (http://metabolights.org) under accession number MTBLS5947.

## Notes

**Phenome, Genome and Environment. Chief Editor**: Colleen Doherty
