# Supplementary figures and images for: Dynamic variation of nutrient absorption, metabolomic and transcriptomic indexes of soybean (Glycine max) seedlings under phosphorus deficiency
Source: AoB Plants. 2023 Apr 10;15(2):plad014. doi: 10.1093/aobpla/plad014 (PMC10132309; doi:10.1093/aobpla/plad014)

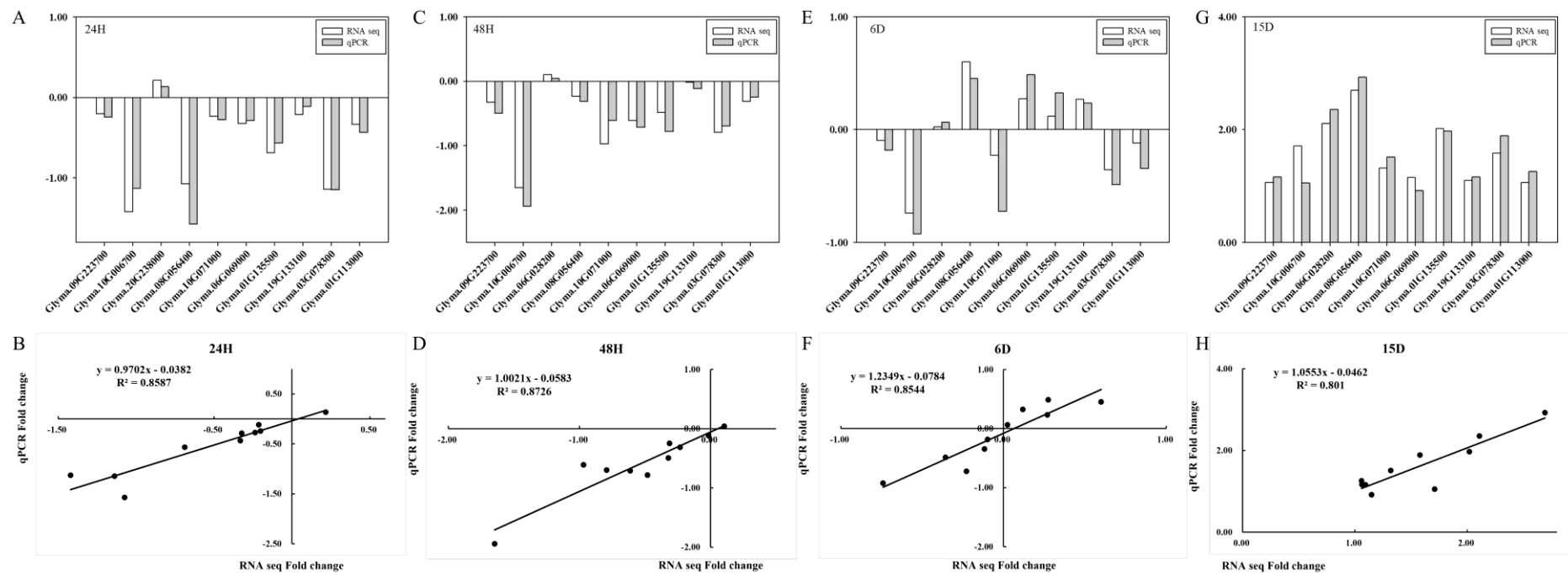

Figure S1. The qRT-PCR of 10 genes in the roots of soybean.

Supplement: plad014_suppl_Supplementary_Figure_S1 [file plad014_suppl_supplementary_figure_s1.pdf]
