# Supplementary material for: Dynamic variation of nutrient absorption, metabolomic and transcriptomic indexes of soybean (Glycine max) seedlings under phosphorus deficiency
Source: AoB Plants. 2023 Apr 10;15(2):plad014. doi: 10.1093/aobpla/plad014 (PMC10132309; doi:10.1093/aobpla/plad014)
Supplement: plad014_suppl_Supplementary_Table_S1 [file plad014_suppl_supplementary_table_s1.pdf]

Table S1. Primers for qRT-PCR of the10 genes in the roots of soybean

Gene	Sense Primer(5’-3’)	Anti-sense Primer(5’-3’)
**reference gene**	GGTGATGTGTTGAAGACTGAA	GTAACTTGAATGTGAGAGGAGAA
**Glyma.09G223700**	GCTCAACTTCTTCCTCCTT	ATCAGAACGATGGCTTGG
**Glyma.10G006700**	GGAGTGGTGGCATTGATT	GCTGTCTCTGGCATCTTC
**Glyma.06G028200**	GCCTTATACTGCTCGTTATC	TGTCTCGGTTCTGTTCAC
**Glyma.08G056400**	TGTTCCAGTTCCAGTTAGAG	TCACCAAGCACCAAGAAG
**Glyma.10G071000**	CCTCCTCCTTCACATCCTA	TTCCATACTCCACCACAGA
**Glyma.06G069000**	CTATTCCACTCAACTCCACTA	GGACACATAACCTTCAACTG
**Glyma.01G135500**	AGAGGAGGACTTCATTATTCG	TGATGTTGCTGTAGTTGACT
**Glyma.19G133100**	GAACCAGCATCAGAGTCAA	ACAGTCAAGGAGCAAGAAG
**Glyma.03G078300**	CTTCTGAGTGTTCCTATTGTC	ATCTTGTTCGCTGCTGTT
**Glyma.01G113000**	CTTCTGAGTGTTCCTATTGTC	ATCTTGTTCGCTGCTGTT
